# Identification of *Onosma visianii* Roots Extract and Purified Shikonin Derivatives as Potential Acaricidal Agents against *Tetranychus urticae*

**DOI:** 10.3390/molecules22061002

**Published:** 2017-06-16

**Authors:** Stefania Sut, Roman Pavela, Vladislav Kolarčik, Loredana Cappellacci, Riccardo Petrelli, Filippo Maggi, Stefano Dall’Acqua, Giovanni Benelli

**Affiliations:** 1 Department of Pharmaceutical and Pharmacological Sciences, University of Padova, Via Marzolo, 35121 Padova, Italy; stefania.sut@unipd.it; 2 Crop Research Institute, Drnovska 507, 161 06, Prague 6, Czech Republic; pavela@vurv.cz; 3 Department of Botany, Institute of Biology and Ecology, Faculty of Science, P.J. Šafárik University, Mánesova 23, 04154 Košice, Slovakia; vladislav.kolarcik@upjs.sk; 4 School of Pharmacy, University of Camerino, Via Sant’Agostino 1, 62032 Camerino, Italy; loredana.cappellacci@unicam.it (L.C.); riccardo.petrelli@unicam.it (R.P.); 5 Department of Agriculture, Food and Environment, University of Pisa, via del Borghetto 80, 56124 Pisa, Italy; benelli.giovanni@gmail.com

**Keywords:** biopesticide, eco-friendly insecticide, *Tetranychus urticae*, *Onosma visianii*, shikonin derivatives

## Abstract

There is an increasing need for the discovery of reliable and eco-friendly pesticides and natural plant-derived products may play a crucial role as source of new active compounds. In this research, a lipophilic extract of *Onosma visianii* roots extract containing 12% of shikonin derivatives demonstrated significant toxicity and inhibition of oviposition against *Tetranychus urticae* mites. Extensive chromatographic separation allowed the isolation of 11 naphthoquinone derivatives that were identified by spectral techniques and were tested against *Tetranychus urticae*. All the isolated compounds presented effects against the considered mite and isobutylshikonin (**1**) and isovalerylshikonin (**2**) were the most active, being valuable model compounds for the study of new anti-mite agents.

## 1. Introduction

There is an urgent need for the discovery of new compounds to be used as pest control agents because the synthetic pesticides currently marketed have harmful effects on human health and the environment. Notably, these products may also kill natural enemies, allowing an exponential increase of pest populations [1]. Therefore, there is a need to discover safe products with low risks for human health and environmental damage. In this framework, research on plant secondary metabolites offers new routes to explore in the search of alternatives to conventional pesticides causing no damage to the environment and non-target organisms [2,3,4,5].

The two-spotted spider mite *T. urticae* (Acari: Tetranychidae) is a polyphagous herbivore feeding on over 1100 species, including over 150 with economic value [6]. Thus, it represents a threat for greenhouse and field crops, with special reference to Solanaceae and Cucurbitaceae species, accounting for about 5% loss in agricultural productivity worldwide [7]. It has been reported that, owing to the global warming, the two-spotted spider mite’s damages in agriculture are expected to increase dramatically in the future because the mite’s development is strongly correlated with high temperatures [8]. An important hallmark of *T. urticae* is its quick capability to develop resistance to synthetic insecticides. Furthermore, some biological features such as high fecundity, fast development, and haplo-diploid sex determination make the two-spotted spider mite able to speed up its development of pesticide resistance [9].

*Onosma visianii* Clem, belonging to the Boraginaceae family, sect. *Haplotricha* Boiss., is a biennial herb occurring in steppic and rocky calcareous sites of Central and Southeastern Europe [10]. The plant is characterized by simple indumentum on green organs, composed of multicellular tubercles, with single setae on the top of tubercle and without asterosetules—short rays radially attached to the base of tubercle. The species is characterized by a sterile rosette of linear-lanceolate densely-setose leaves in first vegetation season; then usually a single reddish, erect, and branched stem with terminal cymes arises from the rosette in the second vegetation season. Flowers are sympetalous and heterochlamydeous, with short pedicels and pale-yellow tubular corolla. Fruits are minutely tuberculate beaked nutlets [11]. Roots are characterized by a showy red bark and are traditionally used to heal wounds and burns in several species of *Onosma* [12]. They are also used in folk veterinary medicine as a feed additive for cattle in Montenegro.

Several members of the Boraginaceae family, such as *Lithospermum erythrorhizon* Siebold & Zucc., *Alkanna tinctoria* (L.) Tausch, *Arnebia euchroma* (Royle) I.M.Johnst., *Echium plantagineum* L. and *O. heterophylla* Griseb. have been extensively investigated from a phytochemical point of view [13]. A review has considered plants of the genus *Onosma*, showing the presence of several classes of secondary metabolites as aliphatic ketones, lipids, naphthazarins, alkaloids, phenolic compounds, flavones and naphthoquinones [14]. A recent paper considered *O. panicolatum* naphthoquinones for their anti-inflammatory potential [15], and some oligomers of alkannin and shikonins obtained from *O. echioides* were studied for wound-healing activity [16]. Specifically related to *O. visianii*, seven naphthoquinones were isolated and studied for antibacterial and cytotoxic activities with significant effects found [17].

Naphthoquinones are oxygen-derivatives of naphthalene, a class of plant secondary metabolites formed on a C_6_–C_4_ skeleton (molecular formula C_10_H_6_O_2_) originating from the shikimate pathway and widespread in several families, including Droseraceae, Juglandaceae, Nepenthaceae, Plumbaginaceae, and Boraginaceae, where they act as allelochemicals and defense against predators [13,18,19].

This group of secondary metabolites has attracted high research attention due to their notable pharmacological activities, which include antimicrobial, anticancer, wound healing, anti-inflammatory, and antithrombotic uses [13,20]. They have also been employed as pigments, dyes, cosmetics, and food additives [21]. The main examples are given by plumbagin, juglone, lawsone, alkannin, and shikonin and its derivatives, with the latter as the most important pigments used commercially [22].

It is worth noting that naphthoquinones are considered as promising candidates for the development of botanical pesticides. As an example, plumbagin and juglone showed important acaricidal and insecticidal effects [20,23]. To date, only a few naphthoquinones have been evaluated for acaricidal activity [24].

Here, the hexane extract of *O. visianii* roots was selected as a source of bioactive naphthoquinone to be studied against the two-spotted spider mite *T. urticae*. Firstly, the activity of *O. visianii* extract was tested on mortality and oviposition of *T. urticae*. Then, the activity of compounds **1**–**11** was also determined and for the most active derivatives (**1**–**2**), deeper investigations were performed allowing the observation of acute and chronic toxicity, as well as the oviposition inhibitory effects against the target mite species. The findings of this work provide new insights into the potential of *O. visianii* as a source of highly effective naphthoquinone derivatives as acaricides.

## 2. Results and Discussion

### 2.1. Bioactivity of Hexane Extract on T. urticae

The application of the hexane extract from *O. visianii* roots with 12% of shikonin derivatives caused significant mortality of *T. urticae* adults (Table 1) as early as 24 h from application, with lethal doses causing 50% (LD_50_) and 90% (LD_90_) inhibition of oviposition, estimated as 83.2 and 112.6 μg·cm^−2^, respectively. However, the dose needed to kill 50% of the adults decreased significantly as a function of time, and on day 5 from application, LD_50_ was more than 30 times lower (2.6 μg·cm^−2^). The effect of the hexane extract on inhibition of oviposition by *T. urticae* females is shown in Table 1. The extract was found to inhibit oviposition, and the LD_50_ (LD_90_) was estimated as 2.4 (43.5) μg·cm^−2^.

### 2.2. Isolation of Naphthoquinones and Structure Elucidation of Constituents

**Figure 1:**
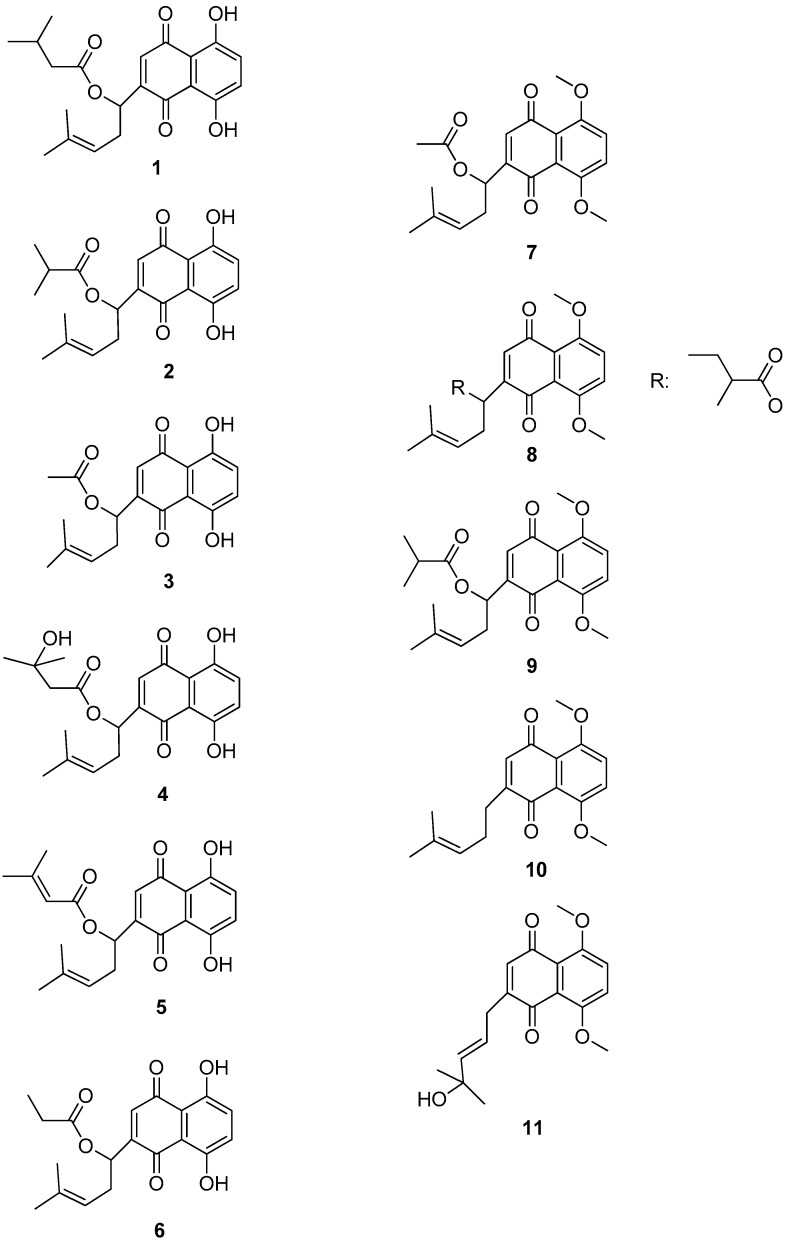
Structure of the eleven naphthoquinones isolated from *O. visianii* roots.

Due to this effect, separation of the constituents of the extract was performed. By extensive chromatographic purification, compounds **1**–**11** were isolated and characterized by the means of spectral methods (Table 2). Among them, two structures (**8** and **11**) were revealed to be new natural products (Figure 1).

Compound **8** was isolated as a yellow solid. The HR-MS spectrum of compound **8** showed the presence of a pseudomolecular ion at *m*/*z* 401.1940, corresponding to the molecular formula of C_23_H_28_O_6_ (calculated 401.1964 Da). The compound presented a single HPLC peak and the ^1^H-NMR spectrum showed signals related to the aromatic part of the compound that was characterized by the presence of singlet at δ 7.32 (H-6/7), 6.60 (H-3), and two identical methoxy groups in positions 5 and 8 (δ 3.96). The ester chain presents a triplet at δ 0.92 (3H, t, *J* = 7.42), and from a correlation spectroscopy (COSY) spectrum the spin system was deduced, and was from H-4′′ (CH_3_ at δ 0.92) to H-3′′ (δ 2.25, CH_2_) from this, later to H-2′′ (δ 2.42, CH), and from this later to the methyl group H-5′′ at δ 1.15 (3H, d, *J* = 7.02). The NMR data support the presence of 2-(1-hydroxy-4-methylpent-3-en-1-yl)-5,8-dimethoxynaphthalene-1,4-dione moiety with ester substituents at OH in position 1′. In particular, the heteronuclear multiple bond coherence (HMBC) correlations from CH_3_-5′′ with carbon resonances at δ 44.8 (C-3′′), 42.4 (C-2′′), and 175.0 (C-1′′) support the presence of a residue of 2-methylbutanoate. Diagnostic HMBC correlations observed from H-2′′′ with carbon resonance at δ 170.0 (C-1′′′) and 23.9 (C-4′′′/5′′′) support the presence of a 3-methylbutanoate residue. Thus, compound **8** was identified as 1-(5,8-dimethoxy-1,4-dioxo-1,4-dihydronaphthalen-2-yl)-4-methylpent-3-en-1-yl 2-methylbutanoate.

**Figure 2:**
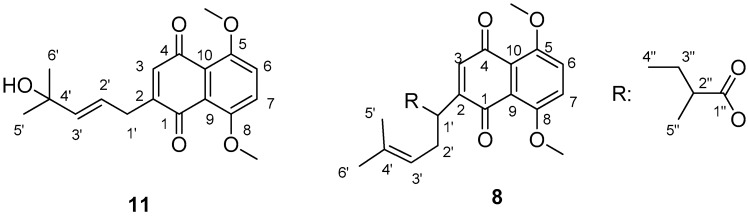
Structure of the new isolated compounds.

Compound **11** was isolated as a yellow solid. The HR-MS spectrum of compound **11** showed the presence of a pseudomolecular ion at *m*/*z* 317.3520 corresponding to molecular formula of C_18_H_20_O_5_ (calculated 317.3563 Da). The ^1^H-NMR spectrum was characterized by the presence of a series of signals ascribable to a naphthoquinone moiety, namely the singlets at δ 7.31 (2H, s), 7.21 (1H, s), partially overlapped to chloroform signal, a doublet at δ 5.80 (1H, d, *J* = 15.85), and a doublet of triplets at δ 5.73 (1H, dt, *J* = 15.85; 5.68) that support the presence of a *trans* olefin linked to a CH_2_, a singlet at δ 3.95 (6H, *s*) supporting the presence of two identical methoxy groups a broad doublet at δ 3.25 (2H, d, *J* = 5.68), and two aliphatic singlets at δ 1.35 and 1.27 (3H each, s) suggesting the presence of two quaternary methyl groups. The heteronuclear single quantum correlation-distortionless enhancement by polarization transfer (HSQC-DEPT) experiment allowed the assignment of the chemical shift of the non-quaternary carbon positions, and revealed the presence in the molecule of four different sp^2^ CH, one sp^3^ CH_2_, and three CH_3_. From the comparison of H, HSQC-DEPT and HMBC the correlations between H and C were deduced and the structure of the compound was assigned. Namely the aromatic portion was assigned to a 1 substituted 5,8-dimethoxynaphthalene-1,4-dione. Diagnostic HMBC correlations were observed from H-6/7 (δ 7.31) with carbon resonances at δ 153.05 (C-5/8), 121.05 (C-6/7), and 100.25 (C-9/10). Further HMBC correlations were observed from H-3 (δ 7.21) with carbon resonances at δ 184.7 (C-1) supporting the presence of keto group, and with a quaternary carbon at δ 148.3 (C-2), suggesting the presence of a substituent in position 2. Considering the signals ascribable to a side chain, HMBC correlations were observed from H-1′ with C-2 and C-1 (δ 184.7), supporting the linkage with the naphthalene moiety at position 2. Further diagnostic HMBC signals were also observed from H-1′ with C-2′ (δ 121.8) and C-3′ (δ 142.1), supporting the presence of a double bond in the side chain. COSY correlation revealed the scalar coupling between H-1′ and H-2′, and from this later to H-3′. The coupling constant of H-3′ (*J* = 15.85) support a *trans* geometry for the double bond. A linkage at position 3′ of a hydroxyl-isopropyl moiety was deduced from the HMBC correlations observed from H-3′ with C-4′ (δ 69.9), C-5′ (δ 30.43), and C-4′. Also, NOESY correlation from H-6′ (δ 1.27) and H-2′ confirm the structure of the side chain as a (*E*)-2-(4-hydroxy-4-methylpent-2-en-1-yl) moiety. Thus, the structure of the compound **11** was assigned to (*E*)-2-(4-hydroxy-4-methylpent-2-en-1-yl)-5,8-dimethoxynaphthalene-1,4-dione. The NMR assignments and the structures of the new isolated compounds are reported in Table 2 and Figure 2, respectively.

### 2.3. Activity of Isolated Compounds against T. urticae

The eleven shikonin derivatives isolated from the root hexane extract (Figure 1) were tested at a dose of 15 μg·cm^−2^, corresponding to the dose of shikonin administered in the test of the crude extract showing LD_50_ and LD_90_. Compounds **1** and **2** caused 100% mortality of the adults and nymphs on day 5 from application (Table 3), and present a value larger than 94% for oviposition inhibition. The other derivatives present significant effects. Despite the relatively low number of isolated compounds, some preliminary structure activity relationships may be observed. Indeed, methoxylation of position 5 and 8 caused significant loss of activity, suggesting an important role of the two *p*-hydroxy groups (C-5 and C-8) for the activity.

Based on analyses of the isolated compounds, isobutyrylshikonin (**1**) and isovalerylshikonin (**2**) were found to provide the highest efficacy. Lethal doses for day 5 from application (Table 4 and Table 5) were determined for these compounds in subsequent acaricidal tests. Isovalerylshikonin was found to provide a significantly higher efficacy, with an LD_50_ (LD_90_) estimated as 1.06 (4.15) and 1.65 (6.67) μg·cm^−2^ for adults and nymphs, respectively (Table 5). These doses were significantly lower compared with isobutyrylshikonin, whose LD_50_ (LD_90_) values were estimated as 2.69 (15.55) and 6.65 (13.16) μg·cm^−2^ for adults and nymphs, respectively (Table 4).

Also, isovalerylshikonin provided a significantly higher inhibition of oviposition (ED_50_ (ED_90_) = 1.15 (2.75) μg·cm^−2^) compared with isobutyrylshikonin, whose ED_50_ (ED_90_) was estimated as 2.71 (9.31) μg·cm^−2^ (Table 6). In addition, both compounds showed ovicidal effects on the eggs of *T. urticae* (Table 7). However, these tests determined a more significant ovicidal effect for isovalerylshikonin (ED_50_ (ED_90_) = 2.1 (5.4) μg·cm^−2^) compared with isobutyrylshikonin (ED_50_ (ED_90_) = 9.7 (60.6) μg·cm^−2^). Thus, as indicated by all the results, a significantly higher acaricidal efficacy was shown by the isolated naphthoquinone isovalerylshikonin.

## 3. Discussion

The activity of quinone-containing compounds against mites can be related to the generation of reactive oxygen species [25], inhibition of mitochondrial respiration [26,27], and DNA intercalation and breakdown. Some shikonins were also active against the stored product pests, *Acanthoscelides obtectus* and *Epilachna varivestis* [28]. Alkannin, the racemic mixture alkannin/shikonin, and acetylated shikonin derivatives have been proved as effective on larvae of the West Nile virus vector *Culex pipiens* [29]. Lawsone synthetic derivatives were highly effective against susceptible and resistant strains of *T. urticae* and *Bemisia tabaci* [30]. 1,4-Naphthoquinone, juglone, 2-methoxy-1,4-naphthoquinone and plumbagin showed antifeedant effects against the cabbage looper, *Trichoplusia ni*, that were higher than those of neem-based products [19]. Plumbagin showed toxicity against the two-spotted spider mite *T. urticae*, the aphids *Myzus persicae* and *Illinoia liriodendra*, and the house fly *Musca domestica* [31]. Acequinocyl, a synthetic compound related to the natural naphthoquinone plumbagin marketed in 1999 [32], is a potent inhibitor of several species of agricultural mites with low effects on beneficial mites, low mammalian toxicity, and short persistence in the environment [33]. The synthetic 2,3-dichloro-1,4-naphthoquinone is registered as an agricultural fungicide that is highly effective against plant pathogens belonging to the genus *Colletotrichum* [34]. 

In the present work, the remarkable acaricidal effects of *O. visianii* root extracts and isolated shikonin derivatives were reported for the first time. The total amount of shikonin derivatives was measured by ^1^H-NMR analysis and was 12% (*w*/*w*) of the dried hexane extract. The activity of the isolated compounds showed similar effects to those observed using the extract. For this reason and for the previous literature report indicating the bioactivity of naphthoquinones, we considered such constituents as the possible active compounds against *T. urticae*. Isolation of compounds and structure elucidation revealed a series of six shikonin esters with hydroxyl and methoxy groups in C5 and C8 position. The observed bioactivity may be in part related to the lipophilicity of these compounds, which allows passage through the insect cuticle and enter into individual cells where they interfere with molting and other physiological processes [2,6]. Our study focused on compounds **1** and **2**, revealing significant effect.

The acaricidal effects of naphthoquinones are due to their capacity to act as potent inhibitors of electron transport [35], as uncouplers of oxidative phosphorylation [36], as DNA intercalating and alkylating agents, and as producers of reactive oxygen radicals [37]. With regard to the latter, the ability of naphthoquinones to generate reactive oxygen species enhances the feeding deterring effects [25], thus playing a pivotal role in the protection of plants against pathogens [18,19]. Also in our experiments on derivatives, the presence of free hydroxyl groups was important for the observed bioactivity. In fact, the derivatives with methoxylation in position 5 and 8 were less active than the corresponding non-substituted compounds.

All the compounds present effects, and the naphthoquinones’ capacities to affect mitochondrial respiration and mite growth and development is a hallmark of many acaricides available on the market, namely rotenone and piericidins [38,39]. In this regard, almost all shikonin derivatives isolated from *O. visianii* are endowed with ester groups. Once they cross the cuticle and enter mite cells, they may undergo breaking of the ester linkage, resulting in the active metabolite which is capable of inhibiting the respiration of mitochondria at Complex III in the electron transfer chain. One of the possible binding sites appeared to be the ubiquinol oxidation site (Q_O_) of Complex III [38].

Finally, it can be noted that the extract from the roots of *O. visianii* provided significant acaricidal efficacy due to the naphthoquinones contained in the extract. Thanks to isolation of individual compounds contained in the extract and their chemical analysis, we successfully determined the most efficient compound—isovalerylshikonin, which showed the most significant acaricidal, antiovipositional, and ovicidal effects against *T. urticae*. The efficacy of the extract and the selected compounds contained in the extract caused chronic mortality which manifested from day 5 after application. However, given that the LD_90_ for the extract as well as for isovalerylshikonin was estimated as less than 10 μg·cm^−2^, which is approximately equivalent to the concentration of 0.1% considering the used application of 10 μL of the application liquid per 1 cm^2^, the acaricidal efficacy of isovalerylshikonin as well as that of the extract from *O. visianii* roots can be considered sufficient for the development of new commercial acaricides.

## 4. Experimentals

### 4.1. Plant Material

Roots of *O. visianii* were collected in the Strážovské vrchy hills, Dolné Vestenice, Rokošské predhorie foothill, Stredná dolina valley, Trenčín Region, Slovakia (48°44′01.0′′ N, 18°23′50.8′′ E, ca. 500 m a.s.l.) in November 2014. Botanical identification was performed by V. Kolarčik, after checking against The Plant List database (www.theplantlist.org). A voucher specimen was deposited in the KO herbarium (Herbarium of the Botanical Garden, P. J. Šafárik University, Košice, Slovakia) with the codex DV13.

### 4.2. Preparation of Extracts

*Onosma visianii* roots were air-dried in the shade at room temperature (~25 °C) for one week and conserved in wrapping papers before extraction. Dry roots were then powdered using a blender MFC DCFH 48 IKA-WERK (Staufen, Germany) equipped with sieves of 2 mm diameter. Eighty grams of root powder were extracted in a Soxhlet apparatus using 500 mL of *n*-hexane. These conditions assured the highest efficiency for extraction of naphthoquinones as reported in the literature [20]. The obtained extracts were concentrated under reduced pressure at 30 °C with a rotary evaporator up to constant weight (yield 2.7% *w*/*w* dry weight). The extract was kept in a glass vial sealed with silicon septa and stored under darkness at −4 °C before chemical analysis and biological experiments.

### 4.3. Isolation and Chemical Analyses

Silica gel plates (cod 5171 Merck) and silica gel (60 mesh) were obtained from Sigma (Milan, Italy). Solvents were obtained from Carlo Erba (Milan, Italy). HPLC Varian 920 chromatograph (Varian, Palto Alto, CA, USA) was used for preparative chromatography. NMR (1D and 2D) spectra were obtained on a Bruker Avance 400 spectrometer (Bruker, Billerica, MA‎, USA). NMR spectra for compounds **8** and **11** can be found at Supplementary Materials. Chemical shifts (δ) are expressed in ppm. The soxhlet hexane extract of *O. visianii* root (2.5 g) was eluted in a silica gel column (5 × 30 cm, 300 g Silica Gel 80 mesh) using cyclohexane (A) and ethyl acetate (B) as the eluting system, starting from 100% A and gradually increasing the B amount up to 50%. Fractions of 12 mL were collected and pooled on the basis of their chromatographic behavior by TLC in 11 different fractions: fr-1 (0.68 g), fr-2 (0.43 g), fr-3 (0.31 g), fr-4 (0.09 g), fr-5 (0.08 g), fr-6 (0.08 g), fr-7 (0.11 g), fr-8 (0.06 g), fr-9 (0.06 g), fr-10 (0.04 g), and fr-11 (0.01 g). All the fractions were used for compound isolation. Further purifications were obtained with semipreparative HPLC on a Zorbax SB C-18 (21.2 × 150 mm, 5 μm) column using as mobile phase methanol and water (0.1% formic acid) in isocratic elution (90:10) for 25 min. The flow rate was 5 mL/min. UV detection was used at 545 and 254 nm. The purity of the isolated compounds was checked by HPLC analysis and was >97% by software integration. Quantification of the shikonin derivatives in the crude extract was performed using ^1^H-NMR as previously described. Briefly, 50 mg of crude extracts were exactly weighed and dissolved in a deuterated chloroform solution of caffeine (1 mg/mL). Peaks were assigned to the methyl groups of caffeine (δ 3.43) and clearly resolved peaks assigned to the H-3 of shikonin derivatives (δ 7.00) were then used for quantitative analysis using a previously published approach [40]. Isolated compounds were isovalerylshikonin **1** (37.2 mg), isobutyrylshikonin **2** (63.2 mg), acetylshikonin **3** (16.6 mg), hydroxyisovalerylshikonin **4** (2.4 mg), shikonin-β,β-dimethylacrylate **5** (58.7 mg), propionylshikonin **6** (1.1 mg), 5,8 dimethoxy acetylshikonin **7** (8.1 mg), 1-(5,8-dimethoxy-1,4-dioxo-1,4-dihydronaphthalen-2-yl)-4-methylpent-3-en-1-yl 2-methylbutanoate **8** (0.8 mg), 5,8 dimethoxy isobutyrylshikonin **9** (0.6 mg), 5,8-*O*-dimethyldeoxyshikonin **10** (4 mg), and (*E*)-2-(4-hydroxy-4-methylpent-2-en-1-yl)-5,8-dimethoxynaphthalene-1,4-dione **11** (0.5 mg). The structures of the compounds were elucidated on the basis of 1D and 2D NMR measurements, comparing obtained data with previously published literature [41,42,43,44].

### 4.4. Mite Rearing

Two-spotted spider mites, *T. urticae* Koch (Acari: Tetranychidae), were obtained from the cultures maintained at the Crop Research Institute (Prague, Czech Republic). The spider mites used in the experiments were reared on bean plants (*Phaseolus vulgaris* L. var. Carmen) in a growth chamber (22–25 °C; a 12 h photoperiod).

### 4.5. Tarsal Toxicity Tests

The method of Pavela [45] was used to determine the acaricidal efficacy of the extract from *O. visianii*. The tarsal test was used to determine the extract efficacy in terms of mortality of *T. urticae* adults after 24 h (considered as acute toxicity) and at 5 days (considered as chronic toxicity) from application. The experiment was done in bean plants (*P. vulgaris* var. Carmen) with discs sized 2 cm^−2^. First, stock solutions of the extract were prepared by dissolving an appropriate amount of the extract in methanol to obtain the concentration series of 1.5%, 1.0%, 0.8%, 0.5%, 0.3%, 0.2%, 0.1%, 0.05%, 0.02%, and 0.01% (*w*/*v*). An automatic pipette was used to uniformly apply 20 μL of the solution on one side of the disc (10 μL·cm^−2^). A concentration series was thereby obtained, equivalent to the doses of 150, 100, 80, 50, 30, 20, 10, 5, 2, and 1 μg·cm^−2^. Only methanol was applied to the control discs. After application, the discs were placed in Petri dishes (5 cm in diameter) with an agar layer 0.3 cm thick on the bottom (to maintain the freshness of the discs and standard ambient humidity).

After evaporation of the solvent (approximately 10 min from application), a fine brush was used to transfer 10 females of *T. urticae* (2–3 days old) on each of the treated sides of the leaf discs. The Petri dishes were placed in a growth chamber (L16:D8, 25 °C). The cut leaf discs were checked after 24 h and 5th day after application, determining the number of dead adults using binoculars. Death was recorded when the larvae did not respond to prodding with forceps. The determined mortality was used to estimate the lethal doses causing 50% (90%) mortality of *T. urticae*. The experiment was repeated 5 times.

### 4.6. Effect on Oviposition

The same method as above was used to prepare bean discs treated with the extract. Five adults (3–4 days old) were transferred using a fine brush on each of the cut bean leaf discs. The cut discs with the adults were placed in Petri dishes with an agar bottom. The Petri discs were placed in a growth chamber (L16:D8, 25 °C). After 24 h, the laid eggs were counted. For female mortality, the number of oviposited eggs was recalculated to live females. The determined number of eggs oviposited by females was used to calculate the percent inhibition of oviposition and to estimate lethal doses causing 50% (90%) inhibition of oviposition compared to the control. The experiment was repeated 5 times.

### 4.7. Acaricidal and Antiovipositional Effect of Compounds Isolated from the Extract

The same method as above, with minor modifications, was used to determine mortality and antioviposition. In order to determine the most active compounds, all 11 compounds were first tested only in the dose of 15 μg·cm^−2^. Each time, 10 adults (2–3 days old) or 20 nymphs (5 days old) were transferred to the treated discs using a fine brush. Mortality of *T. urticae* adults and nymphs was assessed on days 2 and 5 from application. The same method as above was used to determine the inhibition of oviposition for days 2 and 5 from application. For compounds showing 100% mortality on day 5 from application, a series of different doses (13.5, 6.8, 3.4, 1.7, and 0.8 μg·cm^−2^) was used to determine lethal doses causing 50% (90%) mortality or inhibition of oviposition.

### 4.8. Ovicidal Effect

Using a fine brush, we transferred five adults (3–4 days old) onto each 1 cm^−2^ cut bean leaf disc. We placed the cut discs with the adults into Petri dishes having an agar bottom. After 24 h, the adults were taken out and the eggs that had been laid were counted. We left the eggs for 24 h more; then, we used an automatic pipette to uniformly apply 10 μL of methanol containing a specified dissolved amount of the compounds to the cut pieces, so that the concentration series equaled doses of 13.5, 6.8, 3.4, 1.7, and 0.8 μg·cm^−2^. We applied pure methanol to the control discs, which were placed in Petri dishes following application. The Petri dishes were 5 cm in diameter, with an agar layer 0.3 cm thick on the bottom to maintain the freshness of the cut pieces and a standard ambient humidity. We checked the eggs every day for 10 days and recorded the emerged nymphs, which were subsequently removed. Ovicidal efficacy was calculated using the number of eggs that had not emerged 10 days after application. The Petri dishes were put into a growth chamber (L16:D8, 25 °C). We repeated the experiment 5 times.

### 4.9. Statistical Analysis

Experimental testing confirmed that over 20% of the controlled mortality was discharged and repeated. The observed mortality was corrected by Abbott’s formula [46] after the controlled mortality reached 1–20%. The LD_50_ and LD_90_ values and associated 95% confidence limits for each treatment were estimated using probit analysis of dose–mortality data [47].

The following formula was used to determine the inhibition of oviposition (IO):

IO (%) = ((Co − To)/Co) × 100

where To = number of eggs/female in the treated disc, and Co = number of eggs/female in the control disc.

## 5. Conclusions

The present work provided new scientific evidences for the industrial exploitation of naphthoquinones belonging to the shikonin family. In particular, these lipophilic pigments, easily obtainable from the roots of several Boraginaceae species, are promising candidate ingredients to be incorporated in acaricidal products to be used in crop protection.

## Figures and Tables

**Table 1:** Activity and impact on *Tetranychus urticae* oviposition of the *Onosma visianii* root extract.

Activity	LD_50_ (CI_95_) ^a^	LD_90_ (CI_95_) ^a^	Chi ^b^
Acute toxicity (tarsal test, after 24 h, μg·cm^−2^)	83.2 (79.8–89.5)	112.6 (101.5–121.8)	0.158
Chronic toxicity (tarsal test, after 5 days, μg·cm^−3^)	2.6 (1.7–3.5)	9.4 (6.2–15.5)	0.634
Oviposition inhibition (μg·cm^−3^)	2.4 (2.2–3.9)	43.5 (41.8–45.9)	0.525

^a^ LD_50_ and LD_90_ = dose in μg·cm^−2^ causing 50% and 90%, respectively, mortality of *T. urticae* adults; CI_95_ = 95% confidence intervals, the activity is considered significantly different when the 95% CI fail to overlap; ^b^ Chi-square value, not significant at *p* > 0.05 level.

**Table 2:** NMR assignments for the new isolated compounds.

Position	8		11	
	δ_H_	δ_C_	δ_H_	δ_C_
**1**	-	184.7	-	182.8
**2**	-	148.3	-	147.5
**3**	7.21 s	118.9	6.60 t, *J* = 1.22	133.6
**4**	-	184.7	-	182.8
**5**	-	153.1	-	153.4
**6**	7.31 s	121.1	7.32 s	120.8
**7**	7.31 s	121.1	7.32 s	120.8
**8**	-	153.1	-	153.4
**9**	-	121.2	-	120.9
**10**	-	121.2	-	120.9
OCH_3_	3.95 s	57.3	3.96 s	57.8
**1′**	3.25 d, *J* = 5.68	32.0	5.94 m	70.0
**2′**	5.73 dt, *J* = 15.85; 5.68	121.8	2.45–2.60 m	31.1
**3′**	5.80 d, *J* = 15.85	142.1	5.11 m	117.1
**4′**	-	69.9	-	134.9
**5′**	1.35 s	30.4	1.57 brs	14.9
**6′**	1.27 s	26.9	1.64 brs	27.1
**1′′**			-	175.0
**2′′**			2.42 m	42.4
**3′′**			2.25 m	44.8
**4′′**			0.92 t, *J* = 7.32	12.7
**5′′**			1.15 d, *J* = 7.02	17.6
**1′′′**			-	170.0
**2′′′**			2.28 m	44.9
**3′′′**			2.14 m	27.1
**4′′′**			0.96 d, *J* = 6.71	23.9
**5′′′**			0.97 d, *J* = 6.71	23.9

Note: Spectra were acquired at 40,014 MHz for ^1^H and 100 MHz for ^13^C in CDCl_3_ using TMS as internal reference. The chemical shifts (δ) are expressed in ppm. All coupling constants (*J*) are expressed in Hz.

**Table 3:** Activity and impact on *Tetranychus urticae* oviposition of shikonin derivatives from root of *Onosma visianii*.

Compound No.	Mortality ^a^ (%)				Inhibition of Oviposition ^a^ (%)	
	Adults		Nymphs			
	2nd Day	5th Day	2nd Day	5th Day	2nd Day	5th Day
**1**	**48.1 ± 5.3**	**100.0 ± 0.0**	**28.2 ± 5.1**	**100.0 ± 0.0**	**78.7 ± 8.7**	**94.5 ± 8.6**
**2**	**84.1±6.9**	**100.0 ± 0.0**	**84.6 ± 5.8**	**100.0 ± 0.0**	**90.3 ± 3.3**	**97.4 ± 3.5**
**3**	80.1 ± 7.2	91.7 ± 5.8	58.9 ± 7.4	74.3 ± 5.8	81.8 ± 5.8	94.8 ± 7.1
**4**	32.1 ± 3.9	62.5 ± 5.2	58.8 ± 4.6	67.9 ± 4.8	25.4 ± 5.4	52.8 ± 4.9
**5**	20.1 ± 3.5	41.7 ± 2.5	57.9 ± 6.2	80.7 ± 5.3	0.8 ± 0.3	33.1 ± 9.1
**6**	68.2 ± 5.3	89.7 ± 5.2	69.2 ± 7.1	80.7 ± 5.4	64.8 ± 4.8	91.5 ± 9.5
**7**	52.3 ± 4.8	58.4 ± 4.3	84.6 ± 5.3	87.1 ± 7.9	7.2 ± 2.7	48.7 ± 5.7
**8**	40.3 ± 3.7	50.1 ± 3.9	67.2 ± 3.7	69.2 ± 4.8	18.1 ± 8.2	50.1 ± 4.2
**9**	68.2 ± 7.9	79.9 ± 3.8	74.3 ± 5.8	75.3 ± 5.5	66.1 ± 6.1	80.7 ± 6.9
**10**	20.6± 3.9	41.7 ± 3.9	61.5 ± 3.9	68.8 ± 7.2	9.1 ± 1.5	49.5 ± 7.2
**11**	44.4 ± 4.7	66.7 ± 4.8	35.8 ± 7.2	36.4 ± 4.6	34.5 ± 4.5	54.7 ± 8.5

^a^ Mortality (% after correction by Abbott) and oviposition inhibition values are followed by standard deviations (S.D.); all compounds were at 15 μg·cm^−2^; the two most effective compounds are highlighted in bold.

**Table 4:** Activity of isobutyrylshikonin (**1**) on *Tetranychus urticae* mites.

Dose (μg·cm^−2^)	Adults					Nymphs				
	2nd Day ^a^	5th Day ^a^	LD_50_^b^ (CI_95_)	LD_90_^b^ (CI_95_)	Chi ^c^	2nd Day ^a^	5th Day ^a^	LD_50_^b^ (CI_95_)	LD_90_^b^ (CI_95_)	Chi ^c^
13.5	37.9 ± 5.8	92.8 ± 7.3	2.69 (2.25–3.17)	15.55 (11.67–18.93)	5.362	20.8 ± 7.5	94.4 ± 3.8	6.65 (3.95–8.12)	13.16 (12.97–15.89)	2.555
6.8	24.1 ± 7.2	67.8 ± 5.9				12.9 ± 3.2	33.6 ± 7.2			
3.4	31.1 ± 5.9	57.1 ± 3.9				15.1 ± 4.4	21.3 ± 5.9			
1.7	6.9 ± 3.8	35.6 ± 4.9				2.1 ± 0.8	3.3 ± 2.1			
0.8	10.3 ± 5.5	21.4 ± 5.2				0.0 ± 0.0	0.0 ± 0.0			
Control	9.6 ± 0.2	9.6 ± 0.2				16.6 ± 3.3	18.5 ± 1.3			

^a^ Mortality (% after correction by Abbott) of adults or nymphs ± standard deviation (S.D.); ^b^ LD_50_ (LD_90_) = dose in μg·cm^−2^ causing 50% (90%) mortality of adults and nymphs *T. urticae*; CI_95_ = 95% confidence intervals, the activity is considered significantly different when the 95% CI fail to overlap; ^c^ Chi-square value, not significant at *p* > 0.05 level.

**Table 5:** Activity of isovalerylshikonin against *Tetranychus urticae* mites.

Dose (μg·cm^−2^)	Adults					Nymphs				
	2nd Day ^a^	5th Day ^a^	LD_50_^b^ (CI_95_)	LD_90_^b^ (CI_95_)	Chi ^c^	2nd Day ^a^	5th Day ^a^	LD_50_^b^ (CI_95_)	LD_90_^b^ (CI_95_)	Chi ^c^
13.5	75.8 ± 5.8	100.0 ± 0.0	1.06 (0.89–1.26)	4.15 (3.43–5.32)	3.121	20.8 ± 7.5	100.0 ± 0.0	1.65 (0.61–2.82)	6.67 (5.98–8.16)	1.292
6.8	44.9 ± 5.2	96.4 ± 2.6				12.9 ± 3.2	94.4 ± 3.5			
3.4	24.2 ± 3.9	82.1 ± 6.2				15.1 ± 4.4	61.8 ± 3.2			
1.7	20.7 ± 5.1	71.4 ± 5.5				2.1 ± 0.8	48.7 ± 2.1			
0.8	1.1 ± 0.1	39.2 ± 4.9				0.0 ± 0.0	32.2 ± 2.5			
Control	9.6 ± 0.2	9.6 ± 0.2				16.6 ± 3.3	18.5 ± 1.3			

^a^ Mortality (% after correction by Abbott) of adults or nymphs ± standard deviation (S.D.); ^b^ LD_50_ and LD_90_ = dose in μg·cm^−2^ causing 50% and 90% mortality of adults and nymphs *T. urticae*; CI_95_ = 95% confidence intervals, the activity is considered significantly different when the 95% CI fail to overlap; ^c^ Chi-square value, not significant at *p* > 0.05 level.

**Table 6:** Oviposition inhibition activity of isobutyrylshikonin and isovalerylshikonin on *Tetranychus urticae* females.

Dose (μg·cm^−2^)	Isobutyrylshikonin					Isovalerylshikonin				
	Eggs/Female ± SD ^a^	Inhibition Oviposition (% ± SD) ^b^	ED_50_ (CI_95_) ^c^	ED_90_ (CI_95_) ^c^	Chi ^d^	Eggs/Female ± SD ^a^	Inhibition Oviposition (% ± SD) ^b^	ED_50_ (CI_95_) ^c^	ED_90_ (CI_95_) ^c^	Chi ^d^
13.5	2.6 ± 0.3	91.1 ± 3.2	2.71 (1.79–3.96)	9.31 (8.97–15.56)	2.524	0.3 ± 0.1	98.8 ± 2.1	1.15 (0.02–1.29)	2.75 (2.38–3.34)	2.253
6.8	4.3 ± 1.1	85.9 ± 5.5				0.9 ± 0.2	97.0 ± 3.5			
3.4	11.2 ± 0.9	62.7 ± 4.3				2.5 ± 0.8	91.7 ± 2.8			
1.7	19.3 ± 1.1	36.1 ± 4.6				7.5 ± 0.8	75.1 ± 3.1			
0.8	28.4 ± 2.1	5.4 ± 0.9				21.6 ± 1.8	28.1 ± 1.7			
Control	30.1 ± 4.5	-				30.1 ± 4.5	-			

^a^ Average number of eggs laid per female ± standard deviation (S.D.); ^b^ Mean inhibition of oviposition (in %) in comparison with the control ± standard deviation; ^c^ Effective dose ED_50_ (ED_90_) in μg·cm^−2^ causing 50% (90%) inhibition of egg laying by females *T. urticae*, compared with untreated control; CI_95_ = 95% confidence intervals, the activity is considered significantly different when the 95% CI fail to overlap; ^d^ Chi-square value, not significant at *p* > 0.05 level.

**Table 7:** Ovicidal activity of isobutyrylshikonin and isovalerylshikonin against *Tetranychus urticae*.

Dose μg·cm^−2^	Isobutyrylshikonin				Isovalerylshikonin			
	Mortality of Eggs (%) ^a^	ED_50_^b^ (CI_95_)	ED_90_^b^ (CI_95_)	Chi ^c^	Mortality of Eggs (%) ^a^	ED_50_^b^ (CI_95_)	ED_90_^b^ (CI_95_)	Chi ^c^
13.5	58.9 ± 6.5	9.7 (7.8–12.8)	60.6 (58.9–92.2)	1.546	100.0 ± 0.0	2.1 (1.7–2.3)	5.4 (4.5–7.6)	1.478
6.8	43.3 ± 5.5				95.6 ± 7.6			
3.4	18.6 ± 3.9				70.7 ± 3.8			
1.7	10.5 ± 3.6				42.3 ± 5.2			
0.8	5.3 ± 0.8				27.3 ± 5.9			
Control	1.2 ± 0.2				1.2 ± 0.2			

^a^ The average mortality (in %) of eggs after treatment with the compounds isobutyrylshikonin and isovalerylshikonin ± standard deviation (S.D.); ^b^ Effective dose ED_50_ (ED_90_) in μg·cm^−2^ causing 50% (90%) mortality of eggs of *T. urticae*, CI_95_ = 95% confidence intervals, extract activity is considered significantly different when the 95% CI fail to overlap; ^c^ Chi-square value, not significant at *p* > 0.05 level.

## Supplementary Materials

Supplementary Materials are available online.
