# Supplementary material for: Identification of Onosma visianii Roots Extract and Purified Shikonin Derivatives as Potential Acaricidal Agents against Tetranychus urticae
Source: Molecules. 2017 Jun 16;22(6):1002. doi: 10.3390/molecules22061002 (PMC6152756; doi:10.3390/molecules22061002)
Supplement: Supplementary file 1 [file molecules-22-01002-s001.pdf]

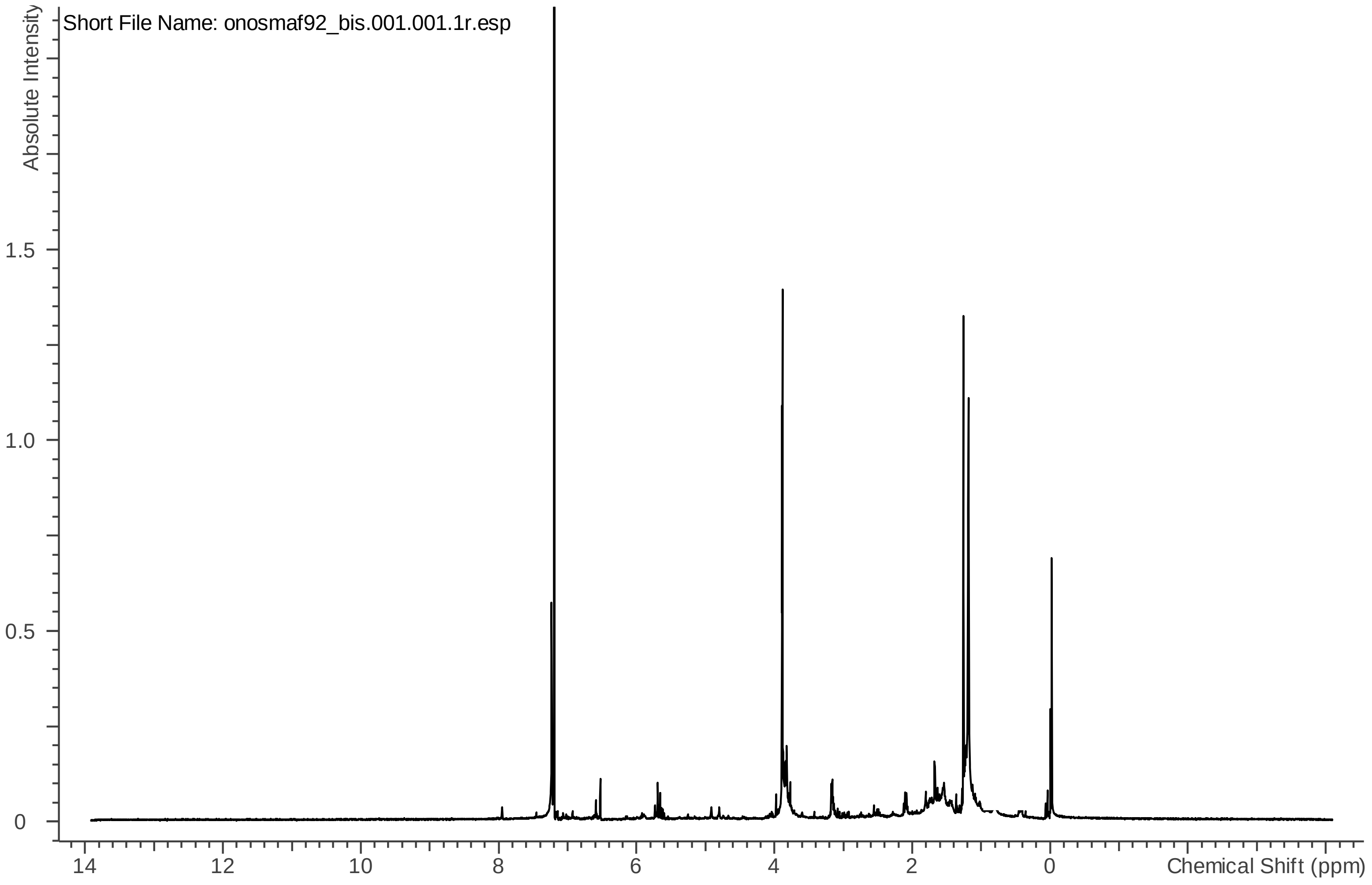

H-NMR of compound 8

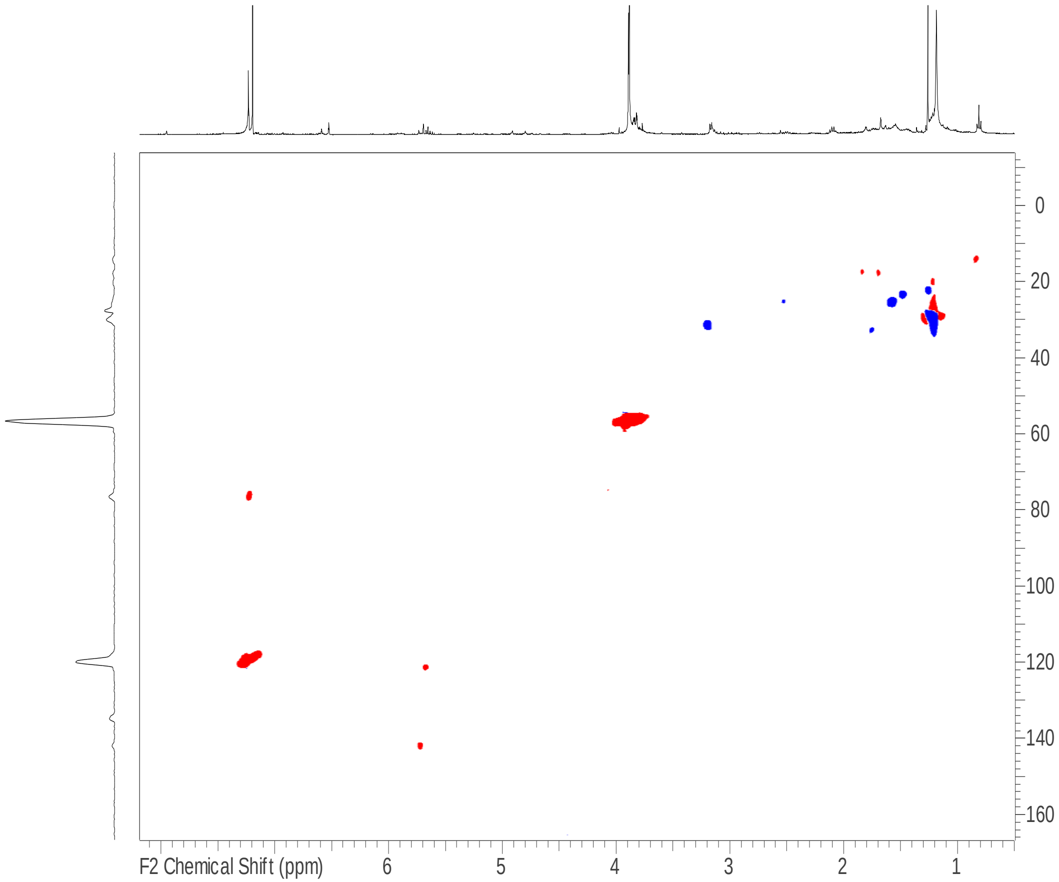

HSQC of compound 8

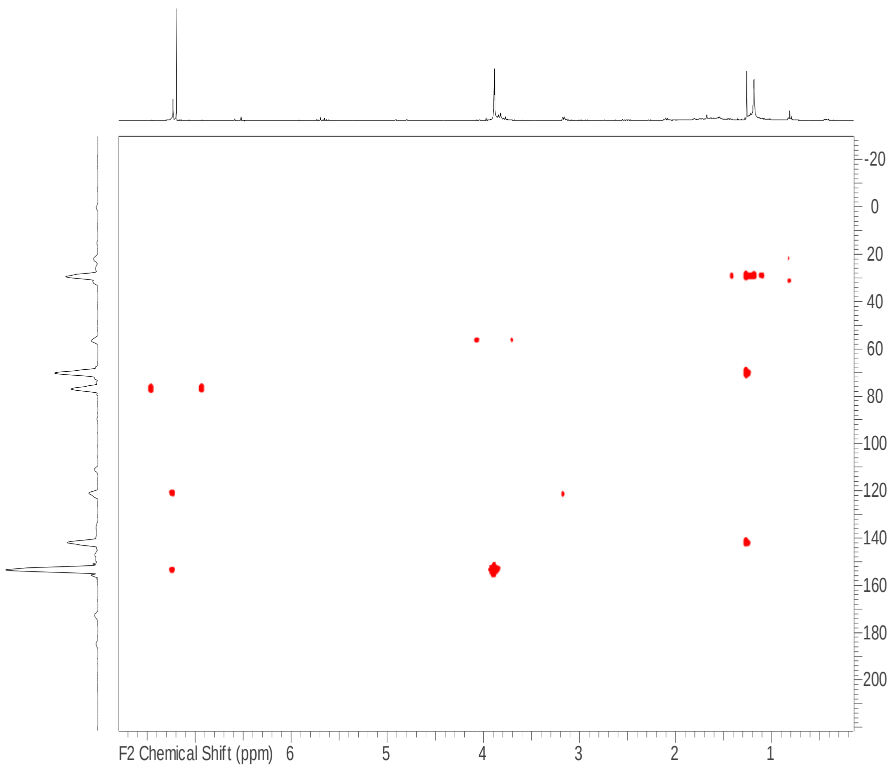

HMBC of compound 8

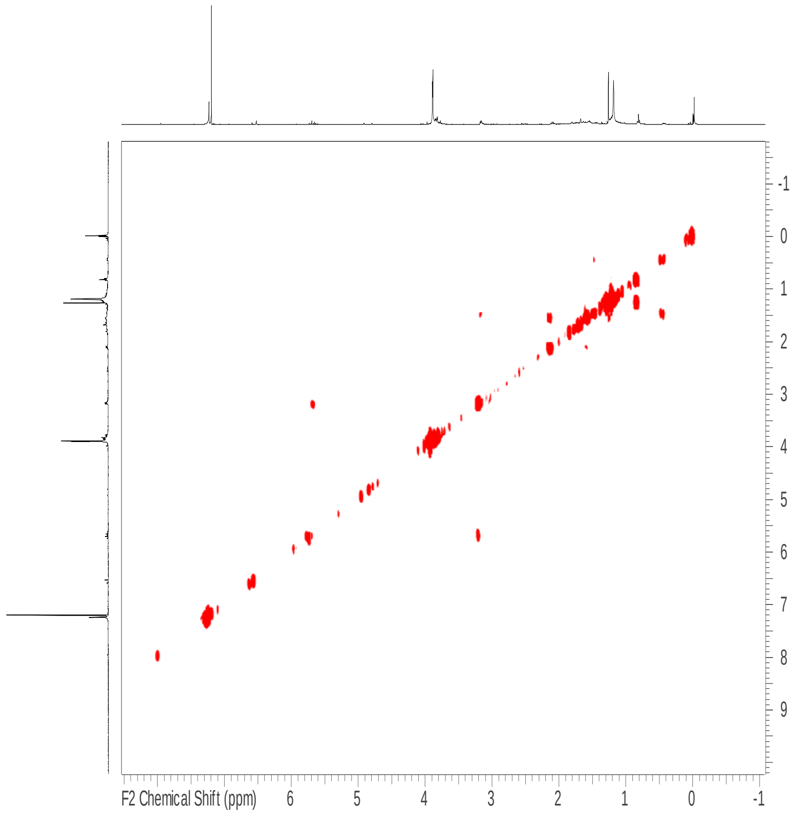

COSY of compound 8

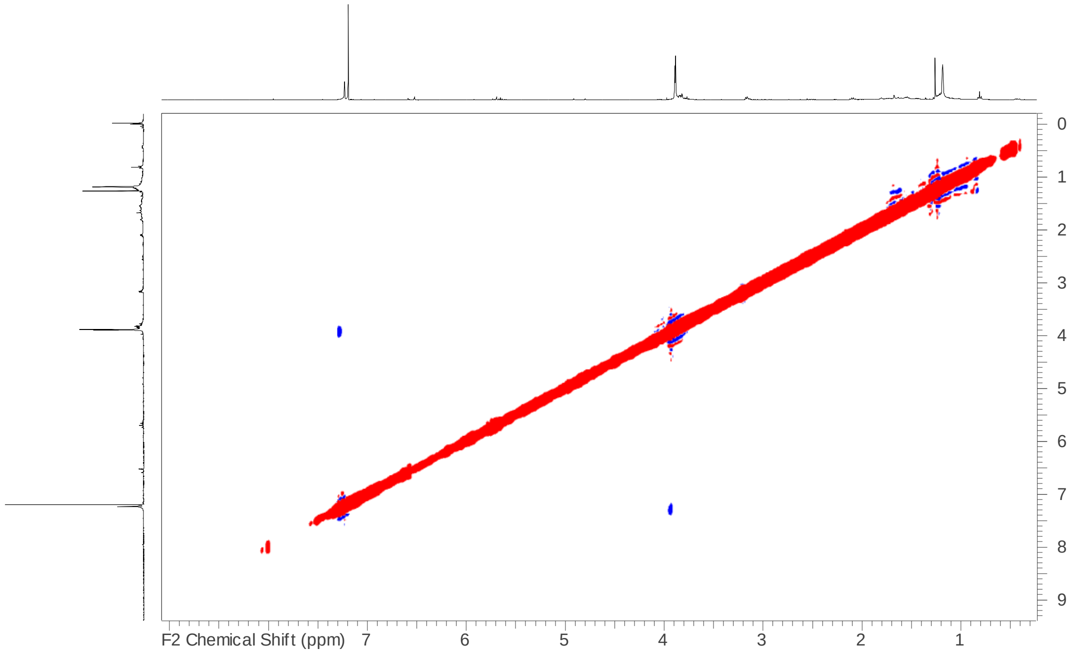

NOESY of compound 8

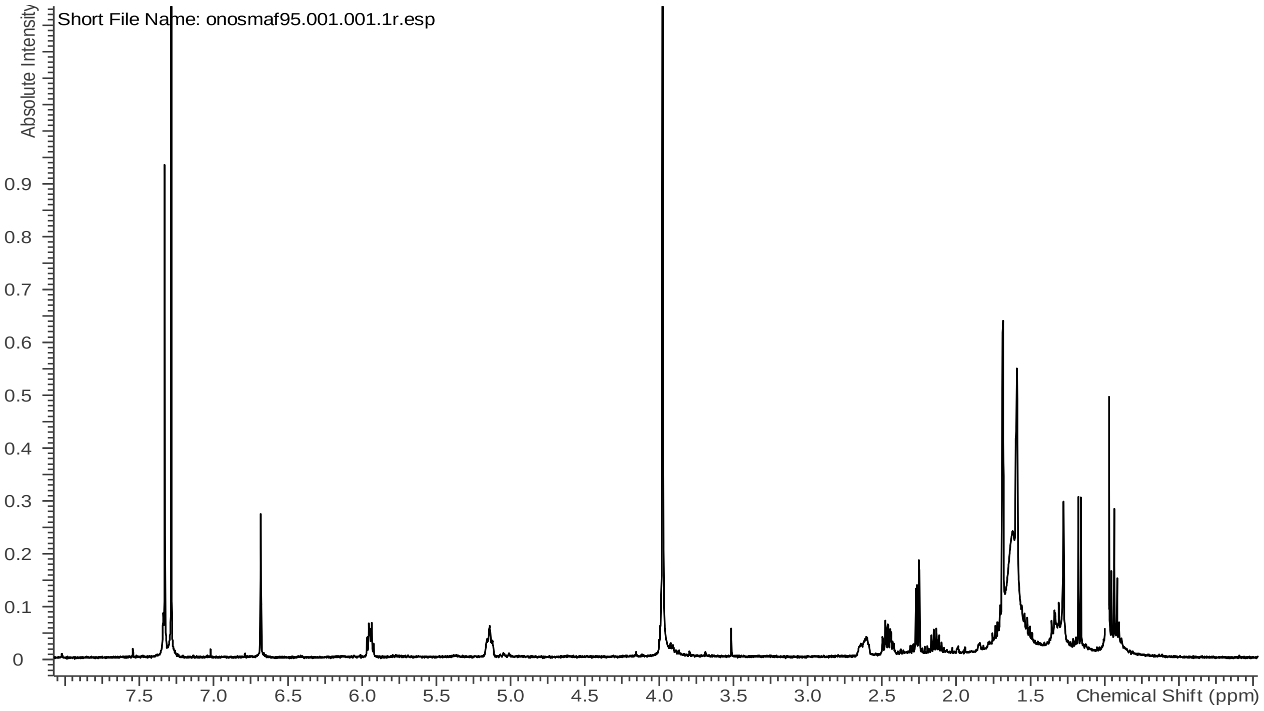

H-NMR of compound 11

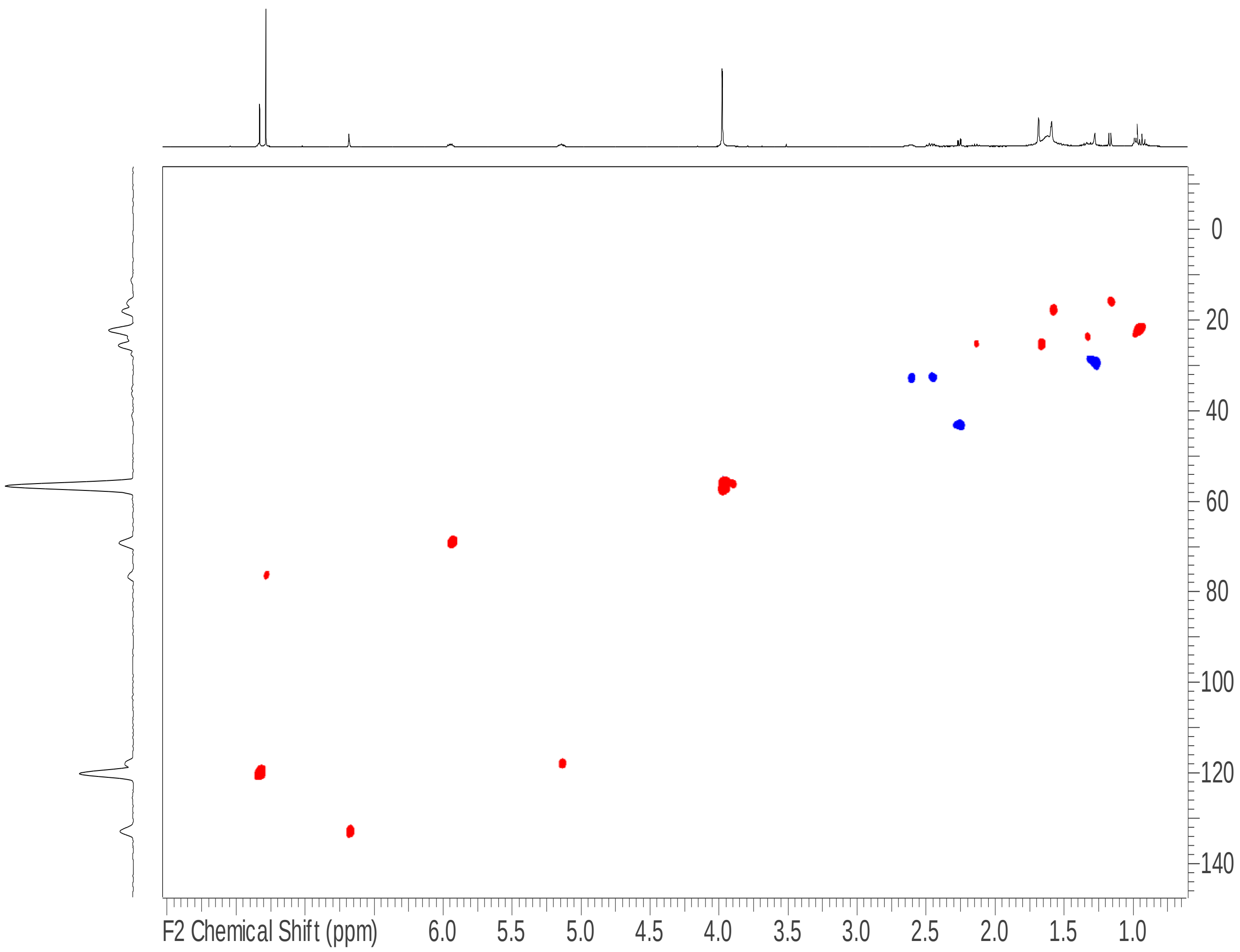

HSQC of compound 11

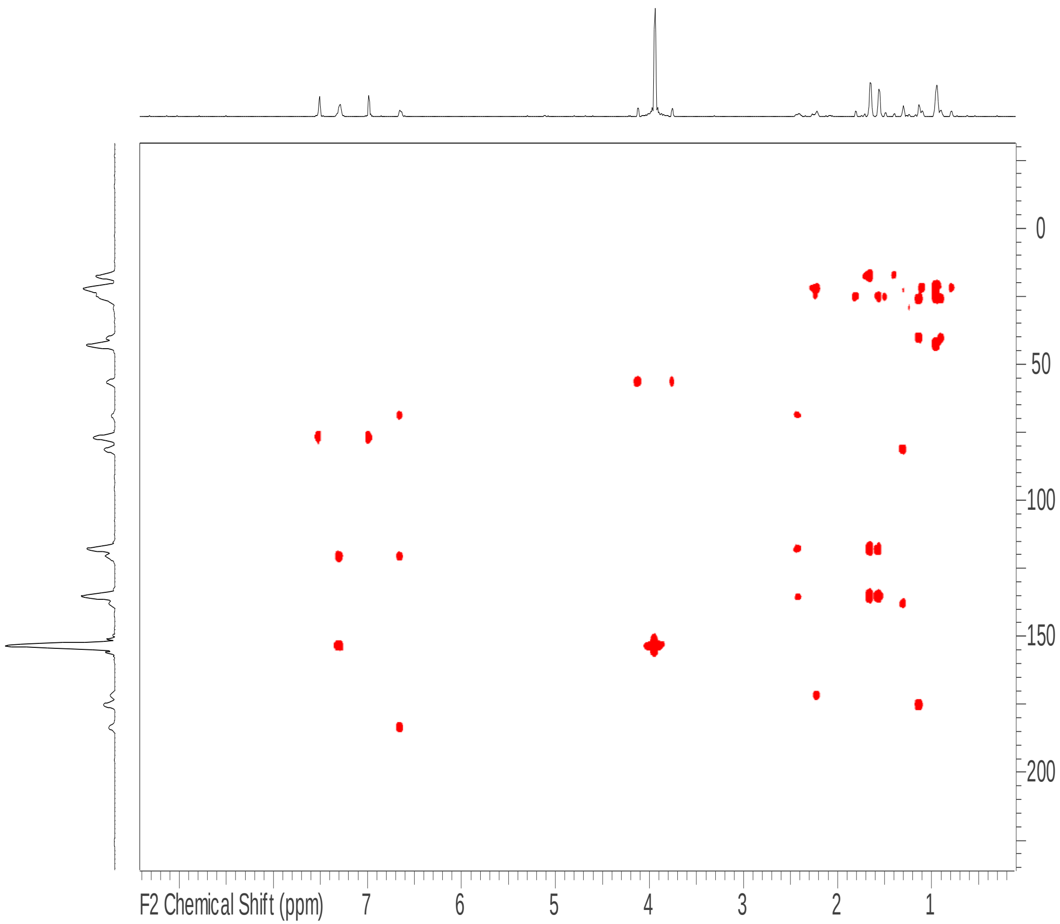

HMBC of compound 11

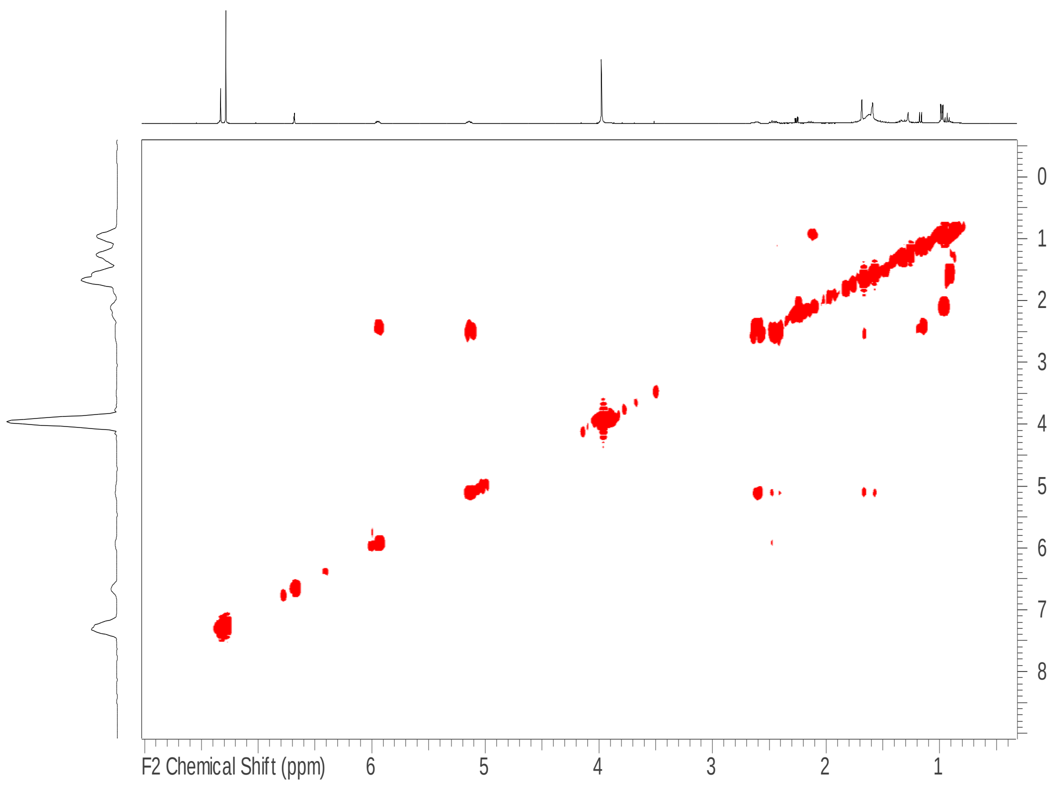

COSY of compound 11

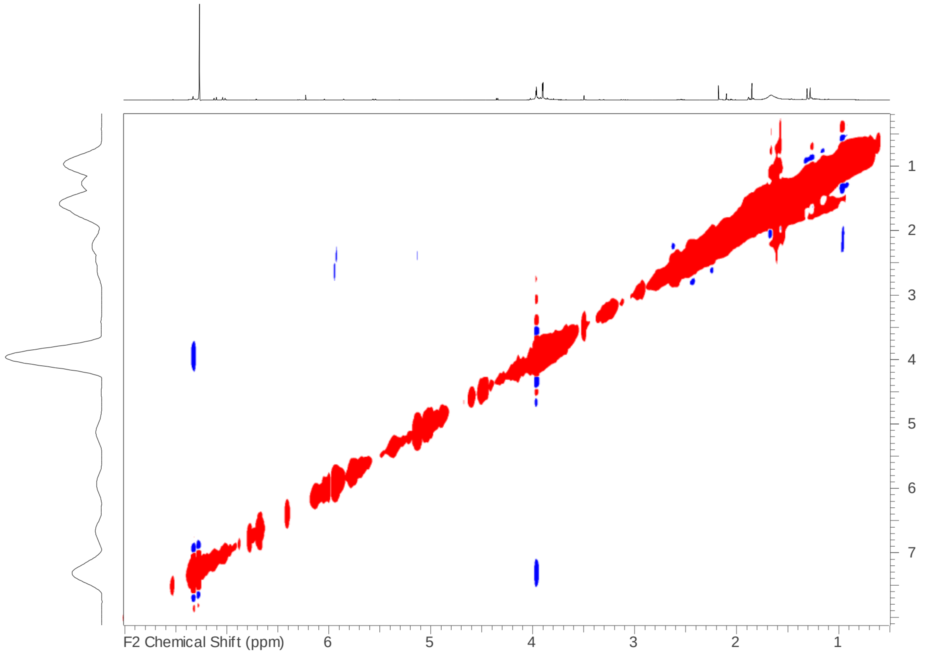

NOESY of compound 11
